# Predicting Conversion from MCI to AD Combining Multi-Modality Data and Based on Molecular Subtype

**DOI:** 10.3390/brainsci11060674

**Published:** 2021-05-21

**Authors:** Hai-Tao Li, Shao-Xun Yuan, Jian-Sheng Wu, Yu Gu, Xiao Sun

**Affiliations:** 1 State Key Laboratory of Bioelectronics, School of Biological Science and Medical Engineering, Southeast University, Nanjing 210096, China; 230169443@seu.edu.cn (H.-T.L.); 230159460@seu.edu.cn (S.-X.Y.); 230198583@seu.edu.cn (Y.G.); 2 School of Geography and Biological Information, Nanjing University of Posts and Telecommunications, Nanjing 210023, China; jansen@njupt.edu.cn

**Keywords:** mild cognitive impairment, Alzheimer’s disease, multi-modality, molecular subtype

## Abstract

Alzheimer’s disease (AD) is a neurodegenerative brain disease in the elderly. Identifying patients with mild cognitive impairment (MCI) who are more likely to progress to AD is a key step in AD prevention. Recent studies have shown that AD is a heterogeneous disease. In this study, we propose a subtyping-based prediction strategy to predict the conversion from MCI to AD in three years according to MCI patient subtypes. Structural magnetic resonance imaging (sMRI) data and multi-omics data, including genotype data and gene expression profiling derived from peripheral blood samples, from 125 MCI patients were used in the Alzheimer’s Disease Neuroimaging Initiative (ADNI)-1 dataset and from 98 MCI patients in the ADNI-GO/2 dataset. A variational Bayes approximation model based on the multiple kernel learning method was constructed to predict whether an MCI patient will progress to AD within three years. In internal fivefold cross-validation within ADNI-1, we achieved an overall AUC of 0.83 (79.20% accuracy, 81.25% sensitivity, 77.92% specificity) compared to the model without subtyping, which achieved an AUC of 0.78 (76.00% accuracy, 77.08% sensitivity, 75.32% specificity). In external validation using ADNI-1 as a training set and ADNI-GO/2 as an independent test set, we attained an AUC of 0.78 (74.49% accuracy, 74.19% sensitivity, 74.63% specificity). Identifying MCI patient subtypes with omics data would improve the accuracy of predicting the conversion from MCI to AD. In addition to evaluating statistics, obtaining the significant sMRI, single nucleotide polymorphism (SNP) and mRNA expression data from peripheral blood of MCI patients is noninvasive and cost-effective for predicting conversion from MCI to AD.

## 1. Introduction

Alzheimer’s disease (AD) is a chronic neurodegenerative brain disease that has no available effective medications or supplemental treatments. Considering the existing circumstances of global aging, the number and proportion of older people will continue to increase. According to statistics [1], approximately 6.2 million people aged 65 or older in the USA will develop AD in 2021 [2] and about 10% of people older than 65 suffer from AD [3]. Therefore, the population of AD patients will also rise sharply in the coming years, which will attract increased public attention.

Mild cognitive impairment (MCI) is known to be the prodromal stage of AD. MCI is a neurological disorder in which an elderly person has mild but measurable changes in cognition. It is worth mentioning that not all people with MCI or in other preclinical stages of Alzheimer’s disease will develop AD [4]. Studies suggest that MCI patients progress to AD at a rate of approximately 10% every year [5]. The goal of this study is to evaluate patients’ characteristics and predict which MCI patients will be more likely to convert from MCI to AD in three years by combining multi-modality data.

More and more studies, such as clinicopathologic [6], atrophy patterns on magnetic resonance imaging (MRI) [7] and amyloid-β fibril polymorphism on solid-state nuclear magnetic resonance (ssNMR) [8], have paid attention to the heterogeneity of AD. Recently, a study categorized 4050 people with late-onset AD into six subtypes according to their clinical cognitive functioning and then utilized genetic data to find the biological differences across these subtypes. The researchers used information on SNPs to construct gene scores for each AD subtype and AD gene scores without subtyping. The gene scores were utilized to predict AD in logistic regression models. The area under the ROC curve (AUC) of AD subtype genetic risk scores for each AD subtype was statistically significantly larger than that of AD gene scores without subtyping [9].

In a previous study, we constructed a method to identify the subtypes of MCI patients based on integrating genetic polymorphism and gene expression data [10]. The results of subtyping patients based on our method have biological and clinical significance. Identifying the subtypes of MCI is critical for implementing precision medicine approaches and ultimately developing successful subtype-specific drugs for AD. In addition, the accurate prediction of progression to AD, particularly in the MCI stage, could potentially offer the possibility to develop treatments to delay or prevent the transition process. Therefore, we constructed a method based on identifying the subtypes of MCI patients to predict whether conversion from MCI to AD will happen in three years, combing sMRI, SNP and gene expression data. If conversion happens within three years, we define these MCI patients as having progressive MCI (P-MCI); otherwise, MCI patients are considered as having stable MCI (S-MCI).

**Figure 1:**
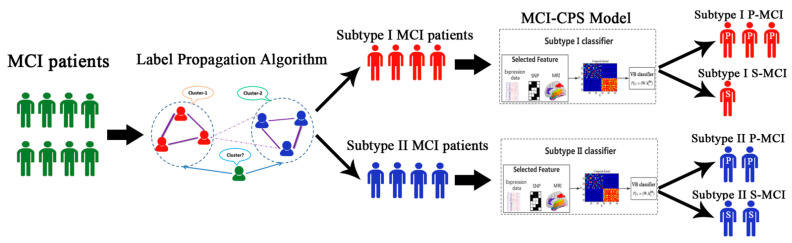
Overview of MCI conversion prediction based on subtyping (MCI-CPS) strategy. Label propagation algorithm is applied to predict subtypes of new patients. Classification of MCI patients in subtype I or II is based on structural magnetic resonance imaging (sMRI), single nucleotide polymorphism (SNP) and mRNA expression data. Multiple kernels learning method is utilized to embed feature sources into the composite kernel. In the construction composite kernel space, MCI patients are classified by variational Bayes approximation algorithm to predict whether conversion from MCI to AD will happen in three years.

Many methods have been used to predict the conversion from MCI to AD [11,12]. However, to our knowledge, the heterogeneity of AD was not taken into consideration among current studies and most of them used invasive biomarkers such as cerebrospinal fluid (CSF). Therefore, in this study, we took advantage of multi-omics data (including genotype data and gene expression profiling of peripheral blood samples from MCI patients) and sMRI data derived from the Alzheimer’s Disease Neuroimaging Initiative (ADNI) [13,14]. We proposed a subtyping-based classification model, called MCI conversion prediction based on subtyping (MCI-CPS), to predict the conversion from MCI to AD. First, we used the subtyping results of our previous research to determine the subtype of each MCI patient. In brief, the similarity network fusion (SNF) algorithm [15] was used to cluster multi-omics data to determine the subtypes of MCI patients in ADNI-1 as a training set and a label propagation algorithm was used to identify the subtypes of MCI patients in ADNI-GO/2 as an independent verification dataset. Then, we utilized the variational Bayes approximation with probabilistic multiple kernel learning (VBpMKL) algorithm [16,17] to build AD risk prediction models for different subtypes and to distinguish between S-MCI and P-MCI patients. Finally, we verified the validity of the model. A flowchart of MCI-CPS is illustrated in Figure 1.

## 2. Materials and Methods

### 2.1. Genomic Data and Imaging Data

Data used in this study were downloaded from ADNI, a multi-site study partnership formed by the National Institute on Aging (NIA), the National Institute of Biomedical Imaging and Bioengineering (NIBIB) and the Food and Drug Administration (FDA) in 2003. This organization is conducting an ongoing longitudinal multicenter study. Its primary goal is to test whether clinical, imaging, genetic and biochemical biomarkers are effective in clinical trials of MCI and AD. The first stage of the initiative, as known as ADNI-1, was completed in 2010 [13]. More up-to-date and detailed information is available at http://adni.loni.usc.edu/ (accessed on 5 December 2020).

In this paper, we used combinations of genetic data (genetic polymorphism and gene expression) and structural magnetic resonance imaging (sMRI) data from the ADNI-1 and ADNI-GO/2 study to predict conversion from MCI to AD.

SNP, gene expression and sMRI data of 125 MCI patients were downloaded from the ADNI-1 study as the training dataset and data on 98 MCI patients were downloaded from ADNI-GO/2 as an independent verification dataset. Both profiles were collected from peripheral blood samples. The two groups of subjects were genotyped using Human 610-Quad BeadChip and Illumina Human Omni Express BeadChip, respectively. Quality control steps were performed on genetic polymorphisms using the PLINK software package (version: release v1.90b.5 [18]. SNPs with missing rate > 0.05, minor allele frequency <0.05 and Hardy–Weinberg equilibrium *p* < 10^−3^ were excluded from the genetic polymorphism set. Then, the SNP data were applied by using the IMPUTE2 program for imputing the missing data with NCBI 1000 Genomes build 37 (UCSC hg19) as the reference panel [19]. The Affymetrix Human Genome U219 Array, which contains 530,467 probes, was used for expression profiling. Thenceforth, we used the R package RMA for normalization of gene expression microarray data [20]. Finally, 49,293 transcripts were retained in this study.

We measured a total of 103 region-of-interest (ROI) volumes, including 35 subcortical structure and 68 cortical volumes, from T1-weighted sMRI images at baseline using FreeSurfer (release 6.0.0) for the two groups of MCI patients. ROI volumes need to be normalized due to the different head sizes of individuals. We used a common normalization method which uses a linear regression model between ROI volumes and intracranial volume (ICV) values to predict ROI-adjusted volume [21]. Adjusted volume is obtained as follows:

(1) $$ROI\_Volume_{adjusted}^{i}=ROI\_Volume_{raw}^{i}-\theta_{i}\left(ICV_{raw}-ICV_{mean}\right)$$

where $ROI\_Volume_{raw}^{i}$ and $ICV_{raw}$ represent the raw volume of the *i*th *ROI* and *ICV* and $\theta_{i}$ is the slope of the regression line between *ICV* and the *i*th FreeSurfer *ROI* volume. $ICV_{mean}$ represents the mean *ICV* volume across all samples. Cortical and subcortical structure volumes range widely and have different dimensions; therefore, we standardized the raw data by z-score.

### 2.2. MCI Subtype Identification Based on Similarity Network Fusion

The similarity network fusion (SNF) algorithm was applied to cluster the MCI patient subtypes, which was processed following previous work [15]. SNF is an integrated characterization method of genomic profiling at multiple levels for subtype identification. The advantage of using SNF is that it is based on complementarity in multiple genomic data types. A brief description of the process is as follows: First, the SNF algorithm uses a similarity measure to construct a patient-by-patient similarity network for each genomic data type. The nodes of the network for each data type represent patients and the weighted edges are equivalent to pairwise sample similarities. Next, the network fusion step updates every network using a nonlinear method, named message-passing theory. After many iterations, multiple networks converge to a fusion network. Finally, based on the spectral clustering method minimize RatioCut, the fusion network was clustered into several subgroups.

Then, we adopted a label propagation algorithm, which is a simple iterative semi-supervised learning algorithm, to predict the subtypes of new MCI patients based on the SNF subtype label. For a detailed description and derivation of the algorithm, please refer to [15,22].

In our previous research [10], we identified two MCI subtypes based on SNF in ADNI-1 and we applied the label propagation algorithm to assign a subtype label to each MCI patient in the ADNI-GO/2 dataset.

### 2.3. Feature Selection Based on Lasso Method

Feature selection is the process of reducing dimension, which can help to improve model performance and prevent overfitting. In this study, feature selection was used to effectively distinguish between S-MCI and P-MCI. We applied a linear model, least absolute shrinkage and selection operator (Lasso) to select a subset of features to distinguish between S-MCI and P-MCI.

Lasso regression analysis is a shrinkage and variable selection method for linear regression models [23]. This algorithm utilizes the L1 penalty for penalization of the regression coefficients, shrinking many trivial features to zero and selects the non-zero variables. In this study, we consider the following L1 regularized minimization problem:

(2) $$\underset{A\in {\mathbb{R}}^{n}}{min}F\left(x\right)=\frac{1}{2}{\left|\left|Ax-y\right|\right|}_{2}^{2}+\lambda {\left|\left|x\right|\right|}_{1}$$

where *A* is a data source matrix, $A\in {\mathbb{R}}^{m\times n}$. m is the number of MCI patients and n is the number of features in each source. The response vector $y\in {\mathbb{R}}^{m}$ represents the labels of MCI patients. *x* is the sparse model we need to learn. *λ* is the regularization parameter and *λ* > 0. Enhanced dual polytope projection (EDPP) is a highly effective, safe screening method that estimates the geometric properties of Lasso regression. It has been proven that EDPP achieves significant speedup for applications. The implementation details of EDPP are available in [24]. We used DPC_package_2.1.1 in the MATLAB toolbox (2013a) to perform the Lasso method [25]. It identifies significant features that are risk factors for the conversion from MCI to AD by the following steps: Set the range to 0.05 < *λ* < 0.5 and use undersampling to prevent an imbalance between positive and negative samples [25]. For each run of Lasso, the number of positive and negative samples is forced to be the same. We ran the inner layer Lasso 1000 times and the outer layer 50 times. After 50 executions, the number of times each feature was selected was accumulated. To identify the optimal number of classification features in the respective classification models, we performed the following experiments on the classifiers for subtypes I and II and the classifier without subtyping. The VBpMKL classifier with fivefold cross-validation repeated 50 times was chosen to classify S-MCI and P-MCI patients in ADNI-1 based on the first n selected feature (*n* = 1, 2, ..., 100) in each data source. The AUC value was used as a criterion to evaluate the number of features for the best classification results.

### 2.4. Construction of the Variational Bayes Classification Model

After feature selection, variational Bayes approximation with probabilistic multiple kernel learning (VBpMKL) classification was implemented to build a prognostic model and predict the conversion from MCI to AD [16,17]. We performed a classification algorithm using the VBpMKL toolkit (http://www.cs.cornell.edu/~damoulas/Site/software.html) running under MATLAB 2013a (accessed on 21 November 2020).

VBpMKL is a Bayesian classification algorithm for integrating multiple feature spaces using a kernel combination methodology, which has been applied to protein fold recognition and disease conversion [26,27]. The kernel method is an effective way to solve nonlinear pattern analysis problems, but a kernel machine consisting of a single kernel function cannot meet practical application requirements such as data heterogeneity or irregularity and sample non-flat distribution, so combining multiple kernel functions to obtain their advantages can achieve better mapping performance. Moreover, typical learning problems often involve heterogeneous or multi-modality data and the multi-kernel approach can provide better flexibility. The concept of variational Bayes is to build an analytical approximation, based on the observed data, to the posterior probability of the set of unobserved variables (parameters and latent variables) [17].

In this study, we made use of the composite kernel, which is a convex linear combination of kernels. The composite kernel construction implements the mean composite kernel, a weighted summation of the base kernels. This method embeds different feature spaces or sources into a kernel space (Hilbert space) using the kernel trick. We defined the N × N composite kernel as

(3) $$K^{\beta \Theta }=\sum_{m=1}^{M}\beta_{m}K^{m\theta_{m}}$$

where *m* is a field of omics and *M* = 3 in this research. β is an *M* × 1 column vector describing each kernel’s contribution and significance and $\theta_{m}$ is a dimensional kernel parameter in all base kernels that indicates the level of smoothing within each kernel.

Subsequently, patients were classified in the construction composite kernel space based on variational Bayes approximation, which is an extension of the expectation-maximization method. More formally,

(4) $$P\left(t_{n}{=m}_{i}{|W,k}_{n}^{\beta \Theta }\right)=\int P\left(t_{n}{=m}_{i}{|y}_{n}\right)P\left(y_{n}{|W,k}_{n}^{\beta \Theta }\right){dy}_{n}{=\varepsilon }_{P(\mu )}\{\prod_{j\neq i}{\Phi (\mu +(w}_{m_{i}}{-w}_{m_{j}}{)k}_{n}^{\beta \Theta })\}$$

where $y_{n}$ is an auxiliary variable, ℰ is the expectation with respect to the standardized normal distribution p(μ)=N(0, 1) and Φ is the cumulative density function. $t_{n}$ is the corresponding label *t* of MCI patient *n*. This formula computes the likelihood that patient *n* belongs to class *i* given kernel matrix $k_{n}^{\beta \Theta }$ data and regression coefficient *W* [16].

After computing the approximate value of the entire posterior distribution of the parameters and latent variables, a classification model was constructed by the VBpMKL algorithm based on this similarity information. For a detailed description and derivation of the algorithm, please refer to [16,26,27].

In order to distinguish between P-MCI and S-MCI for each subtype, we applied the following steps to construct the prediction model (Figure 1). In brief, the prediction model, which contained two subtype-specific classifiers, was constructed based on the multi-modality data. When incorporating a new MCI patient, the label propagation algorithm was used to classify it into the specific subtype and the corresponding subtype-specific classifier was used to predict whether it belonged to P-MCI or S-MCI.

To evaluate the performance of the MCI-CPS model, MCI patients were randomly divided into the training and test sets. In the training set, we used Lasso to select features and the VBpMKL algorithm to construct two subtype-specific classifiers, which predicted the conversion from MCI to AD. In the test set, patients were classified into specific subtypes and conversion was predicted based on the corresponding constructed classifier (Figure S1). Accuracy, sensitivity, specificity and receiver operating characteristic (ROC) curves were utilized as criteria for evaluating the classification performance.

In this study, the classification performance was evaluated via internal and external validation. In the internal validation, we tested the efficiency of the MCI-CPS model with subtyping-based classification in the ADNI-1 dataset using a fivefold cross-validation test. In the external validation, we constructed the MCI-CPS model with two sub-classifiers separately from the ADNI-1 dataset and validated its performance in the ADNI-GO/2 dataset.

## 3. Results

### 3.1. Identifying Subtypes of MCI Patients

In our previous study, we applied the SNF method to identify the subtypes of MCI patients in the ADNI-1 dataset, based on integrating genetic polymorphism and gene expression data [10]. Two MCI subtypes were identified based on our method and were compared by the following factors: the time difference of the conversion from MCI to AD, cognitive scales and sMRI images and significantly enriched pathways based on differentially expressed genes separately. By comparing the sMRI images of subtype I and II MCI patients collected from two-year data, we found that the brain atrophy in subtype I patients was more serious than in subtype II patients. From pathway enrichment analysis, significant pathways of MCI patients in subtype I were mainly related to immune response, while those of subtype II mainly consisted of neuronal signaling-related pathways. The significance of these two subtypes lies in the differences between clinical and biological outcomes, with subtype I having a worse prognosis. The number of MCI patients in subtype I was 61 and in subtype II was 64.

Then, the label propagation algorithm was applied to predict the subtype of any new patient in the ADNI-GO/2 datasets; 34 MCI patients were assigned to subtype I and 64 to subtype II. The distribution information of MCI patients is shown in Tables S1 and S2.

### 3.2. Predicting Conversion from MCI to AD

After identifying the subtypes of MCI patients based on SNP and gene expression data, we constructed classification models for predicting whether the conversion from MCI to AD would happen in three years for the two subtypes separately.

Before constructing the classification algorithm, we selected the significant features in each data source that were relevant to the conversion from MCI to AD. For feature selection, we applied the Lasso linear model to select important traits to distinguish between S-MCI and P-MCI. The optimal number of features in each subtype classifier and without a classifier is shown in Figure S2 and Table S3. After selecting the significant features for each data source, VBpMKL was performed as a classifier to predict the conversion from MCI to AD.

Internal and external validation was conducted as described in the Materials and Methods section. In the internal validation, we first applied fivefold cross-validation to predict conversion within the two subtypes separately based on the ADNI-1 dataset. For subtype I, the training set consisted of 80% of the MCI patients and the test set was the remaining 20%. This procedure was repeated five times, such that five subsets were used as the test set. The same process was used for subtype II. The results show that the area under the ROC curve (AUC) of the sub-classifier in subtype I was 0.8581 and that in subtype II was 0.8623.

To evaluate the classification performance of our subtyping-based prediction strategy, predications were made for the MCI patients in ADNI-1 using a fivefold cross-validation test, named MCI-CPS (5-fold). In detail, the training set consisted of 80% of the MCI patients and the test set was the remaining 20%. The patients in the training set were used to construct two subtype-specific classifiers and those in the test set were classified into the specific subtype using the label propagation algorithm and conversion was predicted based on the corresponding subtype-specific classifiers. This procedure was repeated five times, such that each subset was used as test set. The results show that the AUC of MCI-CPS (5-fold) was 0.8260 (81.82% sensitivity, 77.08% specificity, 80.00% accuracy).

**Figure 2:**
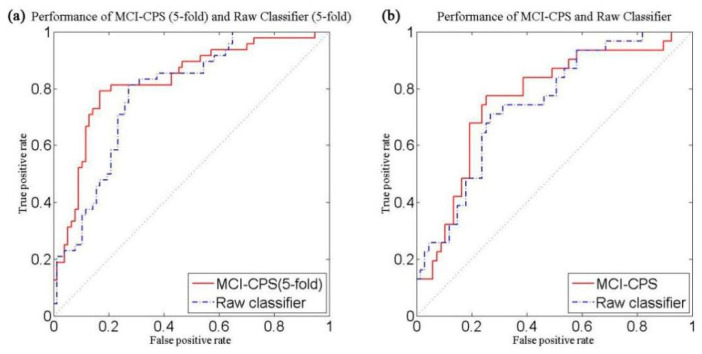
Performance curves for predicting conversion from MCI to AD, comparing MCI-CPS and Raw classifier. (**a**) Five -fold cross-validation in ADNI-1. Solid red line: MCI-CPS (5-fold), AUC = 0.8260; dashed blue line: Raw classifier (5-fold), AUC = 0.7849. (**b**) Performance of model with ADNI-GO/2 as an independent test set. Solid red line: MCI-CPS, AUC = 0.7809; dashed blue line: Raw classifier, AUC = 0.7646.

The raw classifier using VBpMKL directly without subtyping also underwent fivefold cross-validation, which we called Raw classifier (5-fold), for which the AUC was 0.7849 (77.08% sensitivity, 75.32% specificity, 76.00% accuracy). The experimental results are shown in Table 1 and the ROC curve is shown in Figure 2a.

The MCI-CPS (5-fold) test presented remarkably better classification performance than the Raw classifier test based on a variety of classifier evaluation criteria. It was proved that identifying the MCI patient subtypes with omics data improved the accuracy of predicting conversion from MCI to AD.

In the external validation, we used 125 MCI patients in ADNI-1 as the training set and 98 MCI patients in ADNI-GO/2 as an independent test set to further verify the effectiveness of our classification model. Similar to the experimental procedure described above, we constructed the MCI-CPS model and a Raw classifier model without subtyping (Table 2).

As demonstrated in Figure 2b, the prediction result of MCI-CPS showed an AUC of 0.78 compared to Raw classifier, which had an AUC of 0.76.

### 3.3. Important Features

**Figure 3:**
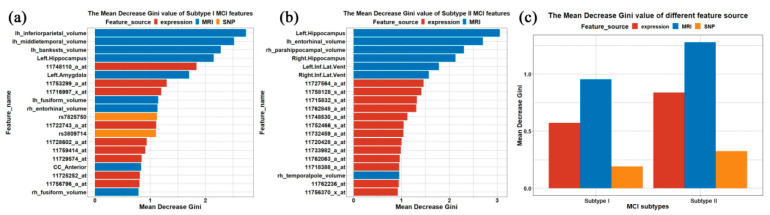
Distribution of mean decrease in Gini coefficient. (**a**,**b**) Gini coefficient of top 20 important features of subtype I and II classifier models. Vertical coordinate indicates name of each feature and horizontal coordinate indicates mean decrease in Gini coefficient. Colors indicate feature sources: red, gene expression; blue, sMRI; yellow, SNP. (**c**) Average Gini coefficient for each feature source in subtypes I and II. Vertical coordinate indicates average Gini coefficient for feature sources of important features of subtype I and II classifier models. Horizontal coordinate indicates MCI subtypes.

We measured and analyzed the importance of the selected features. In this study, feature importance was measured through the mean decrease in Gini coefficient. Briefly, the higher the Gini coefficient, the more difficult it is to take advantage of selected features to divide among categories. Hence, the selection of important features tends to result in a substantial decrease in the average Gini coefficient. We calculated the mean decrease in Gini coefficient for each selected feature of subtype I and II sub-classifiers in order to estimate their important contribution to the classification. Bar plots of the top 20 important variables based on mean decrease in Gini coefficient in each classifier model are shown in Figure 3.

**Figure 4:**
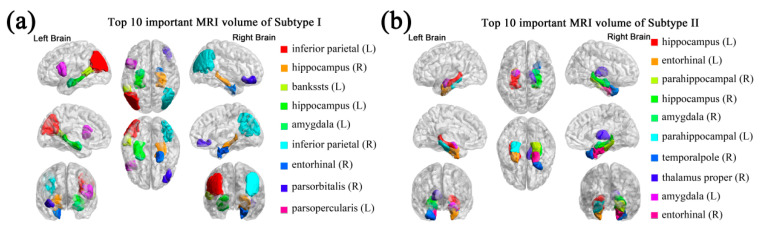
Important sMRI features with an impact on the progression of MCI patients vary across subtypes. Anatomical locations of brain regions with top 10 important sMRI volume features using BrainNet in (**a**) subtype I and (**b**) subtype II. Corpus callosum mid-anterior (CC_Mid_Anterior) in subtype I is not shown.

As seen in Figure 3, sMRI imaging made the greatest predictive contribution to the classification of S-MCI and P-MCI. The anatomical locations of the top 10 important sMRI features are shown in Figure 4 using BrainNet Viewer [28]. The brain regions of the important sMRI features in subtype II are mainly distributed in the temporal lobe, while the important brain regions of subtype I are more dispersed, such as in the parietal, occipital and temporal lobe.

Furthermore, we investigated the impact of sMRI features on the progression of MCI patients with distinct subtypes. A comparison between S-MCI and P-MCI groups based on three representative regional baseline sMRI features in subtypes I and II is shown in Figure S3. We found that certain brain regions showed statistically significant differences between S-MCI and P-MCI in both subtypes (Figure S3a), for example, left and right hippocampus (all *p*-values < 0.01, independent sample *t*-test). Some brain regions showed statistically significant differences between S-MCI and P-MCI only in subtype I (Figure S3b); for example, left and right inferior parietal (*p*-value < 0.01 in subtype I, *p*-value > 0.1 in subtype II, independent sample *t*-test). Some brain regions showed statistically significant differences between S-MCI and P-MCI only in subtype II (Figure S3c), for example, left and right inferior parietal (*p*-value > 0.1 in subtype I, *p*-value < 0.01 in subtype II, independent sample *t*-test), indicating that the important sMRI features that have an impact on the progression of MCI patients vary across subtypes. Thus, classifying MCI patients into meaningful subtypes may allow better targeted treatment to prevent the conversion from MCI to AD.

### 3.4. Comparison with Current Models

**Figure 5:**
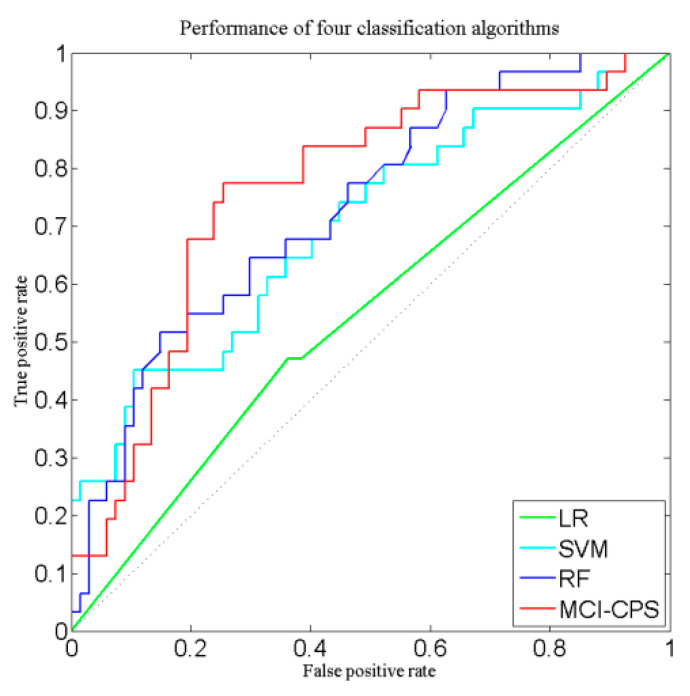
Performance curves of four classification algorithms used for predicting conversion from MCI to AD. Red line indicates VBpMKL, blue line indicates random forest, cyan line indicates SVM and green line indicates logistic regression.

Based on the above study, our subtyping-based prediction strategy got promising results. We also evaluated the performance of different classification algorithms as sub-classifiers in the MCI-CPS model. The VBpMKL algorithm was compared with three common classification algorithms: logistic regression (LR), support vector machine (SVM) and random forest (RF). The classification performance of the four classifiers was evaluated using ADNI-1 as the training set and ADNI-GO/2 as the independent validation set. The classification results are shown in Table 3 and the ROC curves are shown in Figure 5. Comparing these three common classifiers showed that VBpMKL was able to obtain the best classification results.

Furthermore, we compared the performance of our method with current methods for predicting the conversion from MCI to AD. For better comparison, we used recent studies on predicting MCI-to-AD conversion that utilized experimental data from the ADNI datasets with fivefold cross-validation and the results are shown in Table 4.

In recent studies, it has been proved that features from multiple sources share complementary information for disease diagnosis. Naturally, in some methods, features from many different sources or feature spaces are concatenated into longer feature vectors for the purpose of classifying multiple feature spaces. For example, Gao et al. proposed an age-adjusted neural network model that integrated MRI and patients’ age and attained an AUC of 0.81 [29]. Lehallier et al. developed an elastic net algorithm that incorporated MRI, CSF and CICS features and reached an AUC of 0.82 (88% sensitivity) [30]. Westman et al. used a multivariate model on both MRI and CSF measures to predict the conversion from MCI to AD (AUC = 0.76) [31].

More recently, a multiple-kernel method was used for multi-modality data fusion and classification and achieved better performance than the method of concatenating all feature vectors. For instance, Zhang et al. attained an AUC of 0.80 by combining CSF, PET and MRI markers based on multi-task feature selection and the multi-modality support vector machine model [32] and Young et al. proposed a new method using Gaussian process (GP) based on a combination of structural MRI, FDG-PET and CSF to classify P-MCI and S-MCI patients [33].

## 4. Discussion

AD is a neurodegenerative brain disease that has no available effective medications or supplemental treatments. Studies have shown that AD is a heterogeneous disease. Identifying the subtypes of MCI, a prodromal symptom of AD, is critical in order to implement precision medicine approaches and ultimately develop successful subtype-specific drugs. Meanwhile, accurate prediction of progression to Alzheimer’s disease, particularly in the MCI stage, could potentially offer the possibility to develop treatments to delay or prevent the transition process. Classifying MCI patients into meaningful subtypes may provide better targeted treatment to prevent the conversion from MCI to AD.

In this paper, the MCI-CPS model was applied to predict MCI-to-AD conversion in three years using multi-modality data, including structural MRI, genotype data and gene expression profiling, based on patients’ MCI subtype. In a previous study [10], we performed SNF, an integrative clustering of multiple genomic data algorithms, to cluster MCI patients. We conducted experimental studies on subtypes of MCI patients and showed that omics data can define subtypes characterized by biological and clinical significance. In this study, we proposed a subtyping-based prediction model, MCI-CPS, for predicting the conversion from MCI to AD in three years for each subtype separately and achieved satisfactory prediction accuracy. In the internal validation, fivefold cross-validation in ADNI-1, we achieved an overall AUC of 0.83 (79.20% accuracy, 81.25% sensitivity, 77.92% specificity), compared to the model without subtyping, which achieved an AUC of 0.78 (76.00% accuracy, 77.08% sensitivity, 75.32% specificity). In the external validation, using ADNI-1 as a training set and ADNI-GO/2 as an independent test set, we attained an AUC of 0.78 (74.49% accuracy, 74.19% sensitivity, 74.63% specificity).

There are three reasons for these promising results. First, subtype-specific classifiers are more accurate than classification without subtyping (AUC of 0.8581 for subtype I sub-classifier, 0.8623 for subtype II sub-classifier and 0.7849 for raw classifier). Because distinct molecular subtypes of MCI can be identified with different clinical and biological outcomes [10], identifying the MCI patient subtypes will improve the performance of predicting the conversion from MCI to AD [9]. This is important for personalized and precision medicine. Second, it is a natural mechanism to control the greater complexity resulting from the increased dimensionality of multi-modality data, especially omics data [34]. Therefore, we considered that the multiple-kernel method was a more suitable choice rather than simply “concatenating” all modality data together. Third, the multiple-kernel method has more flexibility by using different weights on biomarkers of different sources, while the concatenating method may not be enough for an effective combination of features from different sources [32].

Recently, Park et al. proposed a novel subtyping method for AD according to cortical atrophy of the brain [7]. Among the subtypes identified, areas of brain atrophy with two subtypes, diffuse atrophy and medial temporal predominant, are an unexpected match with the important features of sMRI in subtypes I and II in this study. Therefore, we speculate that when subtype I P-MCI patients convert to AD, they may have the diffuse atrophy AD subtype, while when subtype II P-MCI patients convert to AD, they may have the medial temporal predominant AD subtype.

Currently, the majority of researchers have utilized CSF, PET and MRI data to predict MCI-to-AD conversion, but few methods considered integrating omics data. Most researchers have used single-modality data, such as cerebrospinal fluid (CSF), positron emission computed tomography (PET), or MRI, for predicting MCI conversion to AD. The impact of hippocampal atrophy on behavioral dimensions, such as cognitive deficits, is influenced by the modulation of the personal cognitive reserve, which has significance for the accuracy of clinical diagnosis of AD derived from neuroimaging [35]. For example, Lu et al. introduced a novel deep neural network by solely using FDG-PET metabolism imaging to distinguish S-MCI and P-MCI (accuracy = 81.55%) [36] and Wei et al. applied support vector machine (SVM) with nested cross-validation to classify S-MCI and P-MCI using MRI and obtained an AUC of 0.74 [37]. It is worth mentioning that both SNP and gene expression data came from MCI patients’ peripheral blood. Since the central nervous system can affect the expression of lymphocyte division, neurotransmitters, hormones and other related components in peripheral blood, the expression levels of corresponding genes in peripheral blood are similar to those in brain tissue [38]. Compared to CSF and PET, obtaining biomarkers from peripheral blood is noninvasive and cost-effective for predicting conversion from MCI to AD.

There are several limitations in this study. First, the relatively small size of the dataset limits the interpretation of the results. Large sample sizes and longer follow-up will be required to test the predictive power of classifying MCI types to predict dementia. Further work with larger datasets such as ADNI3, which is a renewal of the ADNI study, is expected to develop better algorithms for practical application. Second, it should be noted that the prediction accuracy of MCI-to-AD conversion in this paper is not as precise as in some published papers (for example, Lu et al.). In the future, combined multi-modality data will improve the accuracy of clinical diagnosis of AD.

Clinical decisions will most likely be dictated by the genetic characteristics of AD patients in the coming years. We believe our method can effectively identify subtypes of MCI patients and can be applied clinically in the future. Tailoring our method based on individual genetic characteristics will help doctors and researchers develop better therapeutic strategies and save many MCI patients from receiving unnecessary toxic therapy.

It is difficult to extract enough features to distinguish S-MCI and P-MCI using single-modality data. In this study, multi-modality data were combined to predict the conversion from MCI to AD. Different types of data share complementary information, which is robust to noise and data heterogeneity [15]. In the future, with the accumulation of clinical data on AD patients, prediction of earlier phase of biology, before the early phase of clinical manifestations, will become possible. A growing number of researchers have found that participation in cognitive leisure activities was an early intervention strategy to reduce the risk of dementia by increasing cognitive reserve [39]. Other types of biological data, such as DNA methylation and miRNA expression, can be integrated to explore biological patterns related to the conversion from MCI to AD. With the addition of human brain tissue expression data, linking SNPs associated with Alzheimer’s disease to their direct effect on gene expression may provide insights into potential drug targets for treatment or prevention of AD.

## 5. Conclusions

In summary, we propose a subtyping-based prediction strategy, named MCI-CPS, to predict the conversion from MCI to AD in three years according to MCI patient subtype. Our model can classify S-MCI and P-MCI patients based on their multi-modal characteristics. In addition, obtaining the significant sMRI, SNP and mRNA expression data from peripheral blood is non-invasive and cost-effective for early recognition of Alzheimer’s disease.

## Figures and Tables

**Table 1:** Comparison of methods for predicting conversion from MCI to AD between MCI-CPS (5-fold) and Raw classifier (5-fold) with 5-fold cross-validation in ADNI-1.

Model	AUC-ROC	Acc (%)	Sn (%)	Sp (%)
VBpMKL (Subtype I)	0.8581	81.97	70.00	93.55
VBpMKL (Subtype II)	0.8623	78.13	88.89	73.91
MCI-CPS (5-fold)	0.8260	79.20	81.25	77.92
Raw classifier	0.7849	76.00	77.08	75.32

AUC, area under ROC curve; Acc, accuracy; Sn, sensitivity; Sp, specificity. First and second rows indicate performance of VBpMKL in subtypes I and II, respectively. Third row represents classification performance of MCI-CPS (5-fold). Fourth row represents classification performance of Raw classifier (5-fold), which was constructed without subtyping.

**Table 2:** Comparison of models for predicting conversion from MCI to AD between MCI-CPS and Raw classifier in ADNI-GO/2 as an independent test set.

Model	AUC-ROC	Acc (%)	Sn (%)	Sp (%)
MCI-CPS	0.7809	74.49	74.19	74.63
Raw classifier	0.7646	69.39	67.74	70.15

**Table 3:** Comparison of methods for predicting conversion from MCI to AD among four classification algorithms derived from ADNI-1.

Method	AUC-ROC	Acc (%)	Sn (%)	Sp (%)
MCI-CPS	0.7809	74.49	74.19	74.63
Logistic regression	0.5541	64.71	71.42	60.00
Support vector machine	0.7005	69.39	48.39	69.39
Random forest	0.7313	70.58	64.28	75.00

**Table 4:** Comparison of methods for predicting conversion from MCI to AD between this study and similar recent studies. First row: MCI-CPS (5-fold), classification after applying SNF method from ADNI-1 dataset. Second row: Raw classifier, model without SNF in ADNI-1. Other rows list performance of compared methods.

Study	Markers	AUC-ROC	Acc (%)	Sn (%)	Sp (%)
MCI-CPS (5-fold)	SNP, mRNA expression data, sMRI	0.83	79.20	81.25	77.92
Raw classifier	SNP, mRNA expression data, sMRI	0.78	76.00	77.08	75.32
Lu et al. (2018)	PET	-	81.55	73.33	83.83
Wei et al. (2016)	sMRI	0.74	66.00	55.30	75.90
Gao et al. (2020)	sMRI, age	0.81	76.00	80.00	73.00
Lehallier et al. (2016)	CSF, sMRI, CICS	0.82	80.00	88.00	70.00
Westman et al. (2012)	sMRI, CSF	0.76	68.50	74.10	63.00
Zhang et al. (2012)	CSF, PET, sMRI	0.80	73.90	68.60	73.60
Young et al. (2013)	PET, sMRI	0.80	74.10	78.70	65.60

AUC, area under ROC curve; Acc, accuracy; Sn, sensitivity; Sp, specificity; sMRI, structural magnetic resonance imaging; PET, positron emission tomography; CSF, cerebrospinal fluid; CICS, Clinical Information and Cognitive Scale.

## Data Availability

Data used in this study are available through the Alzheimer’s Disease Neuroimaging Initiative (ADNI) database (http://adni.loni.usc.edu) (accessed on 5 December 2020).

## Supplementary Materials

The following are available online at https://www.mdpi.com/article/10.3390/brainsci11060674/s1. Figure S1: Flowchart of computational process for constructing a classifier to predict conversion from MCI to AD, Figure S2: Optimal number of classification features, Figure S3: Comparison between S-MCI and P-MCI groups on three representative regional baseline sMRI features in subtypes I and II, Table S1: MCI patient information in ADNI-1, Table S2: MCI patient information in ADNI-GO/2, Table S3: Optimal number of features for each subtype-specific classifier and raw classifier.
