# Supplementary material for: Predicting Conversion from MCI to AD Combining Multi-Modality Data and Based on Molecular Subtype
Source: Brain Sci. 2021 May 21;11(6):674. doi: 10.3390/brainsci11060674 (PMC8224289; doi:10.3390/brainsci11060674)
Supplement: Supplementary file 1 [file brainsci-11-00674-s001.zip › brainsci-1203969-suppl.pdf]

Supplementary Material

Predicting Conversion from MCI to AD Combining Multi-Modality Data and Based on Molecular Subtype

Hai-Tao Li [1], Shao-Xun Yuan [1], Jian-Sheng Wu [2], Yu Gu [1] and Xiao Sun [1,*]

[1] State Key Laboratory of Bioelectronics, School of Biological Science and Medical Engineering, Southeast University, Nanjing 210096, China; 230169443@seu.edu.cn (H.-T.L.); 230159460@seu.edu.cn (S.-X.Y.); 230198583@seu.edu.cn (Y.G.)

[2] School of Geography and Biological Information, Nanjing University of Posts and Telecommunications, Nanjing 210023, China; jansen@njupt.edu.cn

* Correspondence: xsun@seu.edu.cn

Supplementary Figures and Tables

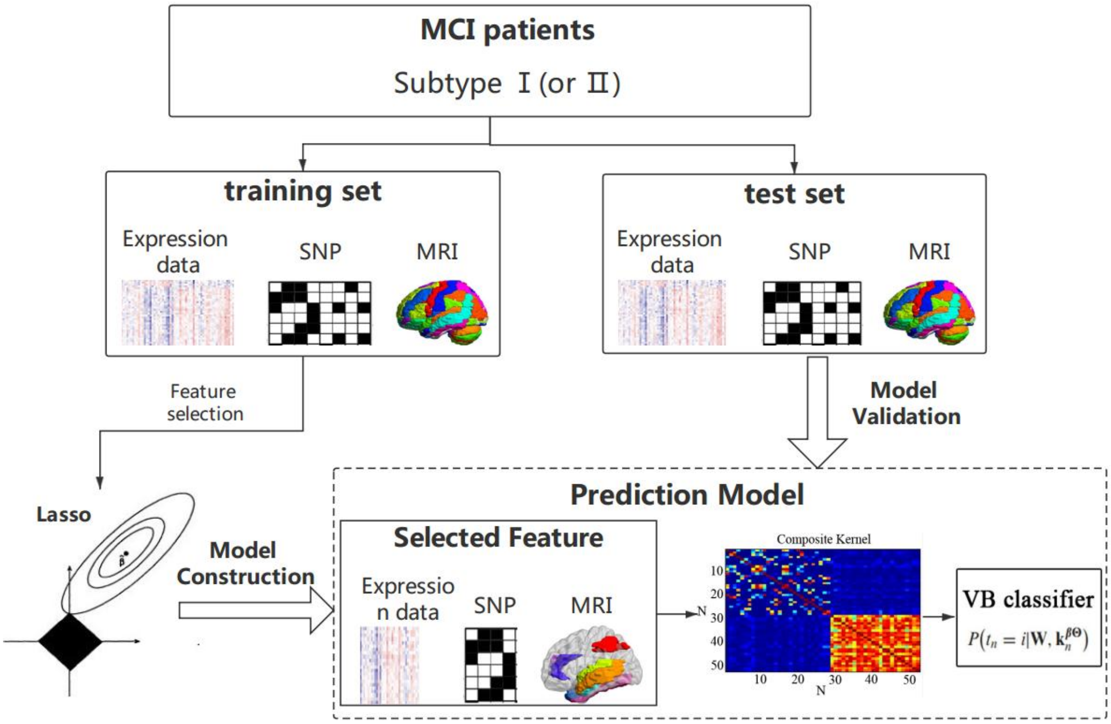

Figure S1. The flow chart of computational process for constructing a classifier to predict the conversion from MCI to AD.

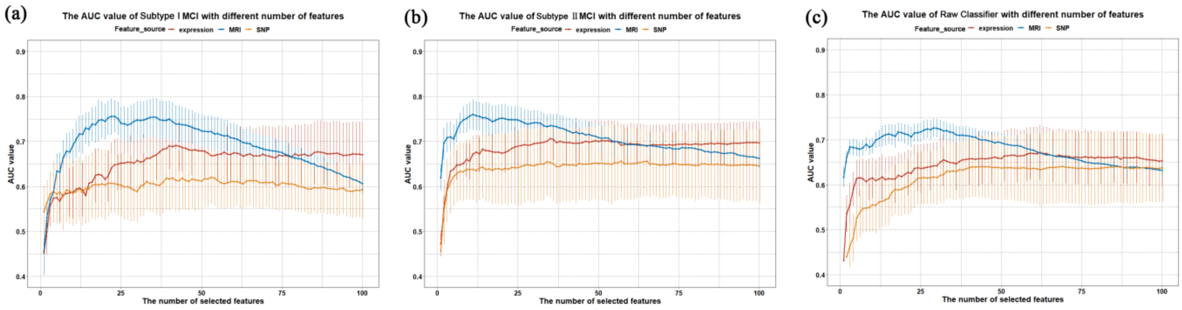

Figure S2. The optimal number of classification features was identified on both Subtype Ⅰ (a), Subtype Ⅱ (b) and classifier without subtyping models (c). The horizontal coordinate indicates the number of selected features. The vertical coordinate indicates AUC value. Red color indicates gene expression data, blue color indicates MRI data, and yellow color indicates SNP.

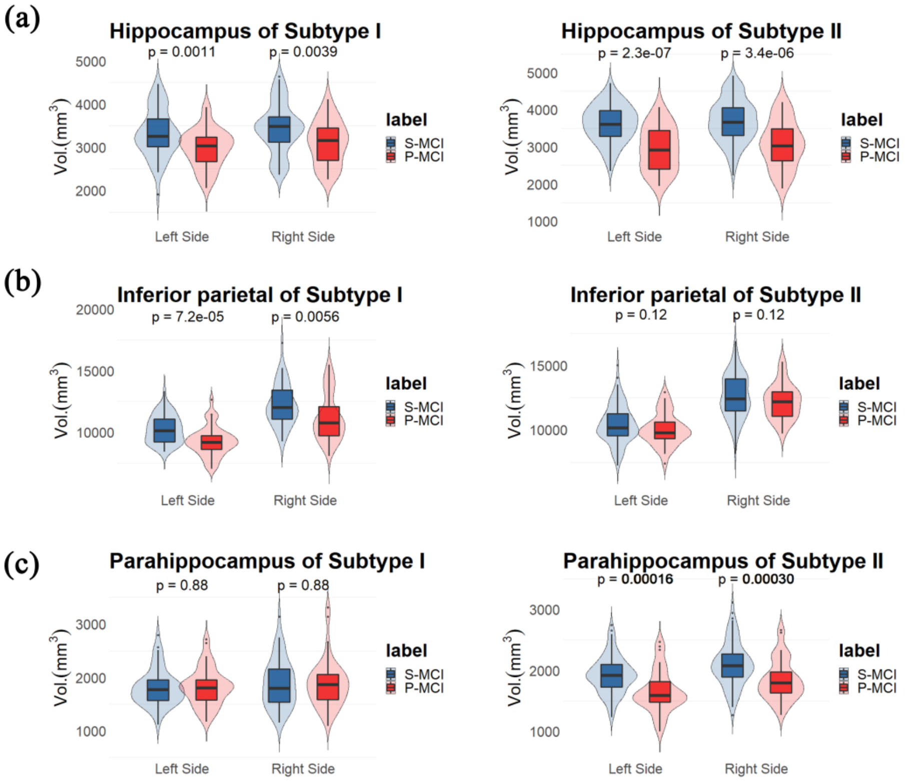

Figure S3. The comparison between S-MCI and P-MCI groups on three representative regional baseline sMRI features in Subtype Ⅰ and Subtype Ⅱ. Comparison between S-MCI (blue) and P-MCI (red) groups on three representative regional baseline sMRI volume features (Hippocampus (a), inferior parietal (b), and parahippocampus (c)) in Subtype Ⅰ (left) and Subtype Ⅱ (right), respectively.

Supplementary Tables

Table S1. The MCI patient's information in ADNI-1.

	Subtype Ⅰ	Subtype Ⅱ	sum
**convert to AD (36th month)**	30	18	48
**not convert to AD (36th month)**	31	46	77
**sum**	61	64	125

Table S2. The MCI patient's information in ADNI-GO/2.

	Subtype Ⅰ	Subtype Ⅱ	sum
**convert to AD (36th month)**	14	17	31
**not convert to AD (36th month)**	20	47	67
**sum**	34	64	98

Table S3. The optimal number of features for each subtype specific classifier and the "Raw classifier" without subtyping, selected by Lasso algorithm.

Study	MRI	SNP	Expression
**Subtype Ⅰ**	23	53	42
**Subtype Ⅱ**	11	57	35
**Raw classifier**	29	70	50
